# OPTimising MEDicine information handover after Discharge (OPTMED-D): protocol for development of a multifaceted intervention and stepped wedge cluster randomised controlled trial

**DOI:** 10.1186/s13063-024-08496-w

**Published:** 2024-09-27

**Authors:** Laetitia Hattingh, Melissa T. Baysari, Holly Foot, Tin Fei Sim, Gerben Keijzers, Mark Morgan, Ian Scott, Richard Norman, Faith Yong, Barbara Mullan, Claire Jackson, Leslie E. Oldfield, Elizabeth Manias

**Affiliations:** 1 Allied Health Research, Gold Coast Health, Southport, QLD 4215 Australia; 2 School of Pharmacy, The University of Queensland, Brisbane, QLD 4102 Australia; 3 School of Pharmacy and Medical Sciences, Griffith University, Gold Coast, QLD 4222 Australia; 4 Biomedical Informatics and Digital Health, School of Medical Sciences, Faculty of Medicine and Health, The University of Sydney, Sydney, NSW 2050 Australia; 5 Curtin Medical School, Curtin University, Bentley, WA 6102 Australia; 6 Emergency Department, Gold Coast Health, Southport, QLD 4215 Australia; 7 Faculty of Health Sciences and Medicine, Bond University, Gold Coast, QLD 4229 Australia; 8 Metro South Digital Health and Informatics, Princess Alexandra Hospital, Woolloongabba, QLD 4102 Australia; 9 School of Population Health, Curtin University, Bentley, WA 6102 Australia; 10 Rural Clinical School, Faculty of Medicine, The University of Queensland, Brisbane, QLD 4350 Australia; 11 Academy of Medical Education, Faculty of Medicine, The University of Queensland, Brisbane, QLD 4006 Australia; 12 The Westmead Institute for Medical Research, The University of Sydney, Sydney, NSW 2145 Australia; 13 General Practice and Primary Care Reform, The University of Queensland, Brisbane, QLD 4072 Australia; 14 School of Nursing and Midwifery, Monash University, Melbourne, VIC 3800 Australia

**Keywords:** Medicine handover, Transition of care, Hospital discharge, Medication-related harm, Community pharmacy

## Abstract

**Background:**

General practitioners (GP) and community pharmacists need information about hospital discharge patients’ medicines to continue their management in the community. This necessitates effective communication, collaboration, and reliable information-sharing. However, such handover is inconsistent, and whilst digital systems are in place to transfer information at transitions of care, these systems are passive and clinicians are not prompted about patients’ transitions. There are also gaps in communication between community pharmacists and GPs. These issues impact patient safety, leading to hospital readmissions and increased healthcare costs.

**Methods:**

A three-phased, multi-method study design is planned to trial a multifaceted intervention to reduce 30-day hospital readmissions. Phase 1 is the co-design of the intervention with stakeholders and end-users; phase 2 is the development of the intervention; phase 3 is a stepped wedge cluster randomised controlled trial with 20 clusters (community pharmacies). Expected intervention components will be a hospital pharmacist navigator, primary care medication management review services, and a digital solution for information sharing. Phase 3 will recruit 10 patients per pharmacy cluster/month to achieve a sample size of 2200 patients powered to detect a 5% absolute reduction in unplanned readmissions from 10% in the control group to 5% in the intervention at 30 days. The randomisation and intervention will occur at the level of the patient’s nominated community pharmacy. Primary analysis will be a comparison of 30-day medication-related hospital readmissions between intervention and control clusters using a mixed effects Poisson regression model with a random effect for cluster (pharmacy) and a fixed effect for each step to account for secular trends.

**Trial registration:**

This trial is registered with the Australian New Zealand Clinical Trials Registry: ACTRN12624000480583p, registered 19 April 2024.

**Supplementary Information:**

The online version contains supplementary material available at 10.1186/s13063-024-08496-w.

## Background

Medication-related harm (MRH) is a significant public health issue, an Australian *National Health Priority*, and the focus of the World Health Organisation’s (WHO) Third Global Patient Safety Challenge [1]. The WHO estimates that medication-related problems cost the economy more than US$42 billion annually, with many of these costs arising during transitions of care [2]. An estimated 250,000 Australian hospital admissions and 400,000 emergency department presentations annually are a direct result of medication-related problems [3]. The post-hospital discharge period is a particularly high-risk period for MRH [4], with a 2018 systematic review showing that 17–51% of older people experience MRH within 30 days of hospital discharge [5]. Furthermore, older people with multiple co-morbidities are at higher risk of readmissions that result from MRH [3, 6–10]. Considering the increase in numbers of older people with multiple co-morbidities and medicines to manage these co-morbidities [7], there is a need to address medicine handover at discharge from hospital to primary care clinicians such as general practitioners (GPs) and community pharmacists. Quality medicine handover necessitates effective communication, collaboration, and reliable information-sharing between the hospital and primary care sectors [5, 11, 12].

Australian standards require hospitals to provide current medicine lists to patients at transitions of care and for the lists to be incorporated into discharge summaries to receiving clinicians [13]. However, studies highlight poor adherence to this activity with inconsistent information transfer from hospitals to GPs and community pharmacists [14–18] and delays in GPs receiving discharge summaries [19, 18]. Ethnographic research identified unclear processes for transferring discharge information and accountability to GPs [20]. GPs may therefore not be informed of patients’ transitions of care journey, relying instead on patients to provide information about their hospital encounters. This lack of communication with GPs contributes to fragmented care and medicine safety risks as delayed, inaccurate, low quality, or incomplete communication from hospitals to GPs contributes to patients’ risk of MRH [11, 12, 21, 22] and hospital readmission [23]. There is also lack of consideration about how patients and families can contribute to enabling effective communication and information-sharing [24, 25].

A 2016 systematic review and meta-analysis showed that pharmacist-led medicine reconciliation programs at hospital discharge are effective in reducing adverse medication-related hospital and emergency department revisits (67% and 28% relative reduction; risk ratio [RR] = 0.33, 95% CI: 0.20–0.53; RR = 0.72, 95% CI: 0.57–0.92 respectively) and all-cause hospital readmissions (19%; RR 0.81, 95% CI: 0.70–0.95) [26]. An Australian randomised trial [27] found that a general practice pharmacist conducting comprehensive medication reviews post-discharge in high-risk patients resulted in a 64% reduction in 30-day, all-cause hospital readmissions and representations (fully adjusted incidence rate ratio = 0.36; 95% CI: 0.15–0.87) [27]. A 2020 systematic review and meta-analysis also showed pharmacist-led interventions with primary care collaboration are effective at reducing readmissions, especially at 30 days follow-up [28]. However, these reductions in hospital admissions are based on a variety of interventions with a need for the standardisation of pharmacist-led medicine reconciliation and transfer of information from hospitals to GPs and community pharmacists.

This brings a need for better integration of digital systems to facilitate medicine information handover at transitions of care [29] and overcoming the current lack of interoperability between software platforms [30, 31] and low uptake and utilisation of digital solutions by clinicians [32, 33]. As a result of these shortcoming, primary care clinicians may not be aware or prompted of patients’ transitions of care or discharge from hospital [32, 33], which is in addition to identified gaps in communication between community pharmacists and GPs [34, 35]. The need for a functional, real-time, secure, and interoperable communication system between GPs and pharmacists is even more important for complex patients with polypharmacy.

## Methods

The proposed study aims to improve medicine handover and digital communication between hospitals, GPs, and community pharmacists when patients are discharged from hospital to primary care. It also aims to increase the uptake of post-discharge medication management reviews by community and credentialed pharmacists. We hypothesise that the co-designed multifaceted intervention will reduce 30-day medication-related hospital readmissions. Secondary outcomes include patients’ understanding of their medicines, quality of life, and health care usage.

Consumer involvement in the study will be aligned with the Guidance for Reporting Involvement of Patients and the Public (GRIPP2) checklist [36]. We followed the SPIRIT criteria [37] in the development of the trial. The stepped wedge cluster randomised controlled trial (SW-CRT) will comply with the extension of the CONSORT 2010 Statement for stepped wedge cluster RCTs [38].

### Study design and setting

A three-phased, multi-method study design will be followed that is underpinned by the Knowledge-to-Action Framework [39, 40]. Ethics approval for phases 1 and 2 was obtained from the Gold Coast Hospital and Health Service (GCHHS) Human Research Ethics Committee on 16 October 2023 (HREC/2023/QGC/101063, GCHealthEthics@health.qld.gov.au). The phase 3 trial was registered with the Australian New Zealand Clinical Trials Registry on 19/04/2024: ACTRN12624000480583p; ethics approval is pending. All items of the World Health Organization Trial Registration Data set are covered in the manuscript.

The multifaceted intervention will involve structured discharge medicine handover from hospitals to community pharmacies where clustering will occur. The community pharmacies are located within the geographical areas serviced by two hospital and health services in South-East Queensland, Australia, namely GCHHS and Metro South Hospital and Health Service (MSHHS), incorporating a mixture of seven tertiary, secondary, and regional hospitals. All study hospitals are part of Queensland Health, the public health service in the state of Queensland, Australia. Queensland Health incorporates 16 hospital and health services. All participating hospitals use a Cerner® electronic medical record system, referred to as the integrated electronic Medical Record (ieMR) system.

We hypothesise our multifaceted intervention will reduce 30-day hospital readmissions due to medication related complications. In addition, we expect our intervention will increase medicine information handover between hospital, GPs, and community pharmacists; increase the uptake of post-discharge medication management review services and medicine reconciliation by community and accredited pharmacists; improve medicine information communication between primary care clinicians; increase patients’ self-reported quality of life and understanding of their medicines and how to take them; and reduce health care usage (regardless of cause).

#### Phase 1: Co-design of intervention with stakeholders and end-users over 9-month period

Key learnings from previous studies [18, 27, 30, 31, 41, 42] and workshops will be used to co-design and integrate the perspectives of hospital and primary care clinicians (GPs, community and credentialed pharmacists), consumers, and other relevant stakeholders. There will be 8–10 1-h co-design workshops with hospital and primary care clinicians, three 2-h workshops with healthcare consumers, and one workshop combining clinicians and consumers. Workshops will be facilitated by two experienced team members (LH and LO), and participants will be invited to share ideas on potential solutions to enhance medicine handover during transfer from hospital, including suggestions on a potential digital solution.

#### Phase 2: Development of the intervention over 12-month period

Building on the results of the phase 1 workshops, this phase will develop infrastructure and resources to streamline medicine information handover from hospital to the community setting. This phase involves four key steps to ensure success: developing handover guidelines, developing and pilot testing a digital solution, customising the intervention, and defining the clusters for the phase 3 trial.

We have partnered with the Pharmaceutical Society of Australia (PSA, the peak Australian pharmacist professional organisation), established software vendors, key stakeholders (hospital and primary care clinicians, managers and department heads), and healthcare consumers to identify, modify, or develop a digital solution based on the user requirements identified in the co-design phase. The digital solution will be pilot tested and refined with end-users: around five general practices and five community pharmacies and one of the participating hospitals [43]. Scenario-based usability testing followed by a debrief interview will allow for qualitative feedback to determine intervention usability and acceptability. In concert, we will collaborate with healthcare consumers and the PSA to develop guidelines and training modules to upskill community pharmacy staff in transitions of care, including the new digital solution.

Retrospective hospital data will be used to identify postcodes within the catchment areas of the two hospital and health services that contain populations of community dwelling patients who have a high prevalence of hospital readmission within 30 days after discharge from a previous admission. These data will be used to identify clusters of community pharmacies that are located within these ‘high-risk’ (of readmission) populations.

#### Phase 3: Stepped wedge cluster randomised controlled trial (SW-CRT) of newly designed intervention over 12-month period

**Fig. 1:**
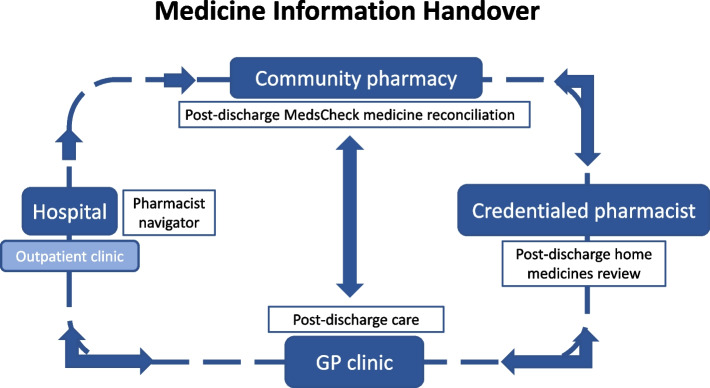
Multifaceted intervention

We will perform a 12-month SW-RCT [44], comparing the intervention with standard care. Twenty clusters of community pharmacies will be pragmatically allocated to receive the intervention. Data will be collected from all clusters over the study period. Each cluster will provide before and after intervention data. This study design is represented in Fig. 1. The anticipated date of first enrolment is 1 October 2025, and last day for recruitment is 30 September 2026.

**Fig. 2:**
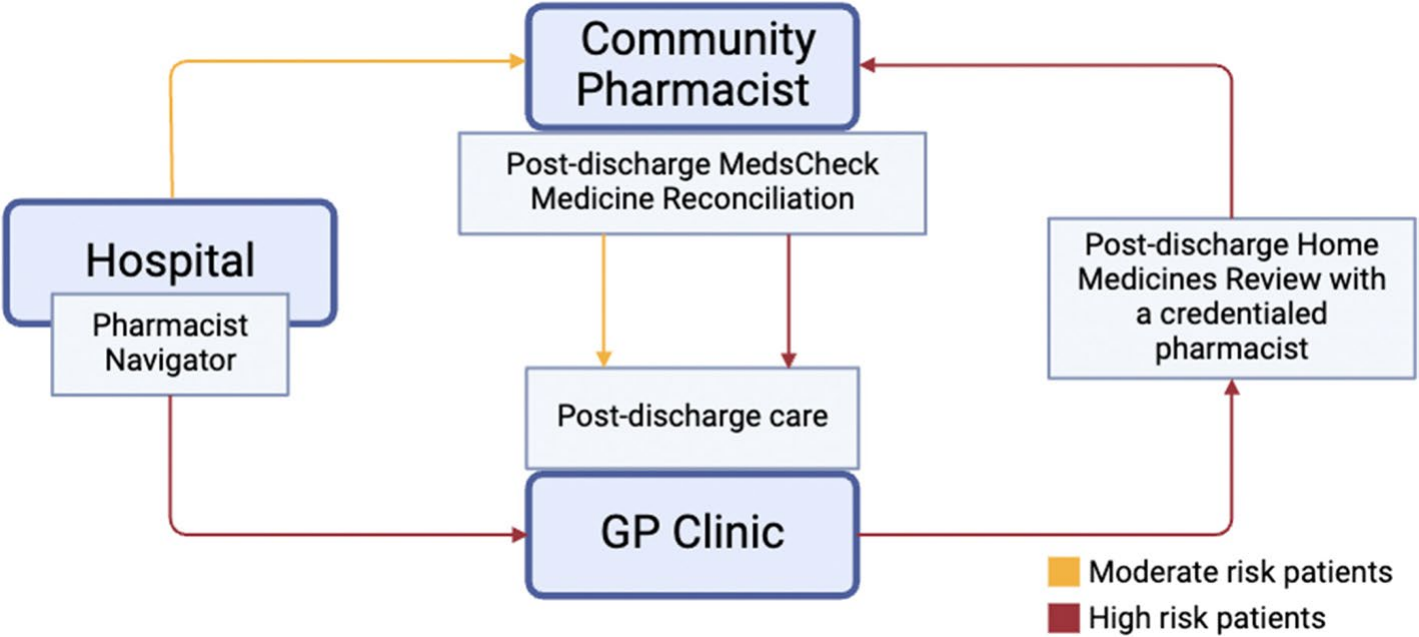
Provision of medication management review services

**Fig. 3:**
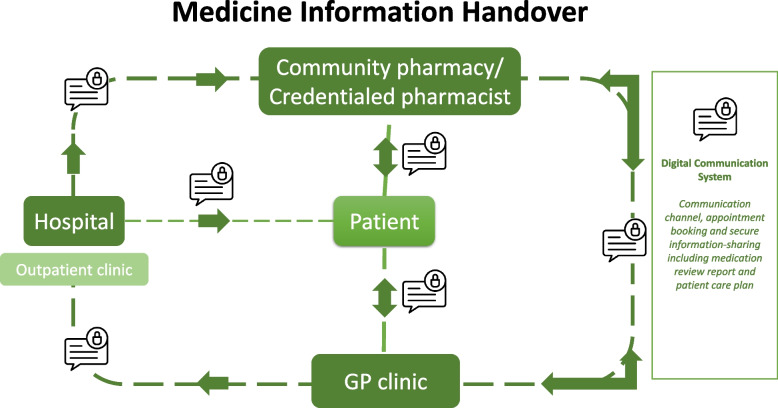
Digital solution

A hospital *pharmacist navigator* will facilitate a medicine handover at each of the participating hospitals. Navigators will work with the patient to connect with their nominated community pharmacy and GP. Using established medication management review *(MMR) services*, the patient’s medicines will be reconciled at the primary care level in the community pharmacy or in the patient’s home and follow-up actions communicated to the GP (Fig. 2). An innovative *digital solution* (Fig. 3) will link hospitals, community pharmacists, GPs, and patients together and, through asynchronous communication, provide all parties with information on all actions being performed throughout the transition of care.

The SW-CRT design will be used to assess the effects of the multifaceted intervention over a 30-day follow-up period following hospital discharge. A 1-month lead in phase is included, where the pharmacy cluster is not considered as being in either the control or intervention phase, and the data collected during this time will not contribute to the final outcome analysis. The SW-CRT design will enable the intervention to be provided at every community pharmacy cluster by the end of the study period, to measure possible underlying temporal trends (such as seasonal variation in admissions) and to prevent potential direct/indirect educational effects of the intervention carrying over to the control phase (which precludes a crossover design). Phase 3 will incorporate an evaluation of the impact of the intervention on key outcomes, a process evaluation, and an economic evaluation.

### Phase 3 sample size

The phase 3 sample size was calculated for the primary outcome, taking into account the intracluster correlation coefficient (ICC), the expected baseline number of readmissions, effect of the intervention, and the desired power of the study (power = 80%, *α* = 0.05) [44, 45]. Patterns of ICCs were drawn from other sources [27, 46] to assume an ICC of 0.15. Based on previous studies [27, 28], it is estimated there will be a reduction in unplanned readmissions from 10% in the control group to 5% in the intervention at 30 days. With an expected 20% drop out rate and aiming for 90% power, we aim to recruit 10 patients per pharmacy cluster per month which will provide a total sample size of 2200 (excluding the cluster lead-in period).

### Study participants

Phase 1 workshop participants will be purposively selected hospital clinicians (doctors, pharmacists and nurses), primary care clinicians (GPs, community and credentialed pharmacists), and healthcare consumers, all having discharge medicine handover experience as either a clinician or end-user. Participants will be selected to ensure maximum variation (age, professional experience, role, gender). Allocation of participants to each group will be done in advance to avoid power imbalances [47].

Phase 2 will involve purposively selected hospital and primary care clinicians, managers and department heads, and software vendors to develop the intervention and handover guidelines/material for hospital and primary care clinicians. The digital solution will be pilot tested on selected general practices, community pharmacies, and a participating hospital.

The Australian Institute of Health and Welfare census data will be used to obtain population data for the areas serviced by the various hospitals to determine the number of suburbs for each hospital service area. Phase 3 will identify community pharmacy clusters whose population catchments, based on the suburb postcodes, capture community dwelling patients with high rates of 30-day readmission to hospital. Pharmacies and GP practices within those clusters will be invited into the study. Only pharmacies who consent to being involved will be recruited into the study. Once all pharmacies have consented, each pharmacy hub will be randomised to a different step in the stepped wedge design which will determine how long each hub will be in the control and intervention phase. The pharmacist navigator at each hospital will triage patients at risk of hospital readmission due to potential MRH by means of a risk stratification processes, use of local electronic medication record dashboards, and liaison with inpatient unit/ward pharmacists, nurse navigators, and other hospital clinicians.

Patients at risk of hospital readmission (according to the study developed risk stratification process) will be approached to be recruited to the study through the pharmacist navigator at each participating hospital.

Inclusion criteria:

- Ability to understand and the willingness to sign a written informed consent document
- Age ≥ 18 years

Exclusion criteria:

- Receiving chemotherapy
- Discharging to an aged care facility
- Receiving palliative care

**Fig. 4:**
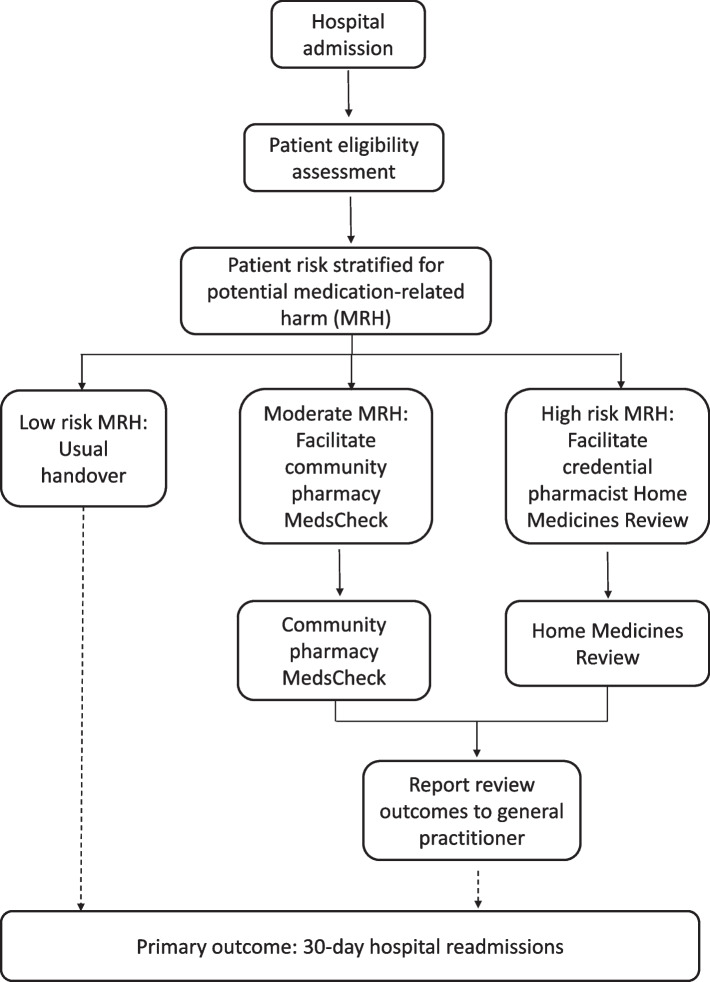
Intervention flow diagram

**Fig. 5:**
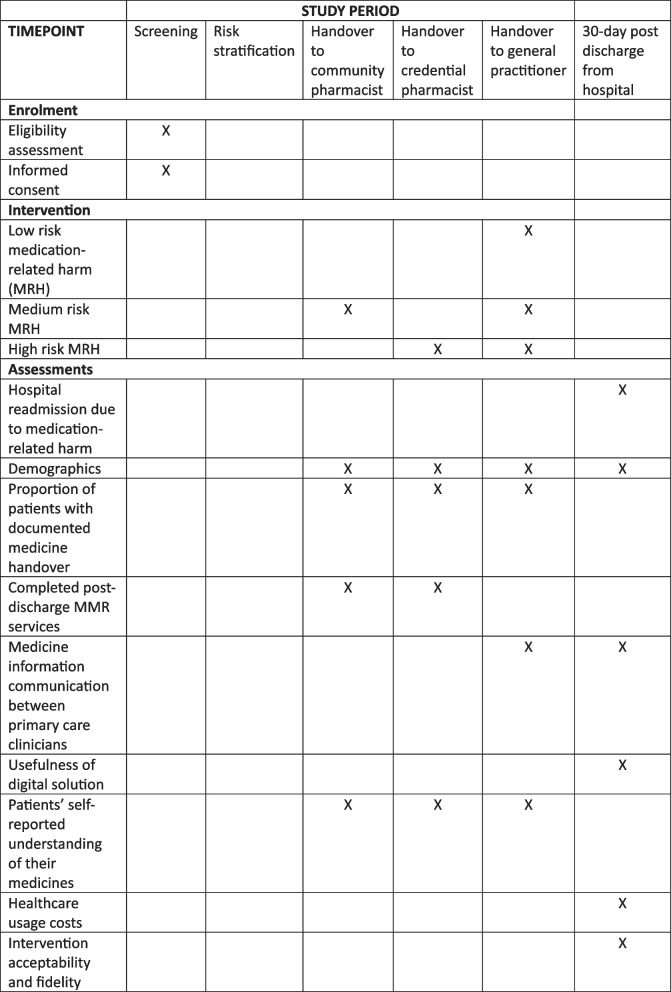
Trial SPIRIT [37] figure

The pharmacist navigator will use the risk stratification information to facilitate a structured handover of medicine information to patients’ community pharmacies, GPs, and credentialed pharmacists to trigger appropriate MMR services (Figs. 4 and 5).

There is no anticipated harm and compensation for trial participation.

### Study outcomes

#### Primary outcome

The primary outcome for the study is a comparison between intervention and control patients of (mean and median) unplanned hospital readmissions at 30-days post-discharge due to medicine-related harm as defined and ascertained below.

Hospital readmission events will be collected from the electronic medical record systems of the seven trial hospitals. The indexed admission will be any inpatient admission via any pathway (e.g. emergency department, elective surgery, outpatients) with an unscheduled representation to the emergency department within 30 days of discharge from the indexed admission. A panel consisting of four senior clinicians (emergency physician, geriatrician, specialist in general medicine, pharmacist) will consider each readmission event to determine if it is possibly medication related and assess its causality, severity, and preventability as MRH [48–50]. The panel will be blinded as to whether the readmission event came from an intervention or control cluster. Each event will be recorded by considering:

- What was the medicine?
- What was the event?
- Has the participant experienced MRH (doubtful, possible, probable or definite) [49]?

MRH will be defined as harm from non-adherence to a prescribed medicine, unintentional medicine error, or adverse medicine reaction [5, 51] within 30 days post-discharge from hospital. It will exclude intentional overdose or misuse but will include unintentional overdoses or misuse. Where the patient is considered to have experienced MRH (definite, probably and possible), the panel will further consider:

- Was the MRH preventable (definite preventable, possibly preventable, not preventable or unable to evaluate) [48, 49]?
- What was the severity (fatal, life-threatening, serious or significant) [50, 52]?
- What was the main cause (adverse drug reaction, unintentional error, non-adherence) [52]?

#### Secondary outcomes

1. Comparison between intervention and control patients (mean and median) of the proportion of patients with documented medicine handover in hospital electronic medical records when patients are discharged from hospital to primary care; data 3 months post completion of intervention of patients with a documented pharmacist and/or GP follow-up appointment organised prior to discharge from hospital through hospital medical records and follow-up
2. Comparison between intervention and control patients (mean and median) of completed post-discharge MMR services and medicine reconciliation by community and credentialed pharmacists; data 3 months post completion of intervention through self-reported survey of primary care pharmacists
3. Mode of medicine information communication (i.e. phone, fax, email, etc.) between primary care clinicians; data 3 months post completion of intervention through GP practice and pharmacy notes and survey of primary care pharmacists
4. Use of digital solution for medicine handover communication from hospital to primary care clinicians and between primary care clinicians; data 3 months post completion of intervention through digital data records
5. Patients’ self-reported understanding of their medicines, how to take them and quality of life; telephone survey within 30 days post-discharge of patients (control and intervention groups; mean and median) incorporating EQ-5D-5L [53] questions, hospital service utilisation since discharge, self-reported understanding of medicines, and feedback on the multifaceted intervention
6. Healthcare usage economic analysis through a stepped cost-effectiveness/cost-utility analysis that will evaluate the implementation of the navigator, the MMR, and the digital medicine handover solution as a single package of care. The costs of the package of care will include the salary costs of the navigator and of the pharmacist conducting MMR services, costed using standard pay scales and including on-costs. The cost of the digital medicine handover solution will be considered in terms of the cost of rolling it out more broadly (i.e. development costs will be noted but not included in the economic evaluation itself). The cost of readmissions in both arms will be included, potentially offsetting some of the package cost with data from the participating hospitals and/or diagnosis-related group information. As 30-day readmissions are the primary focus of the clinical evaluation, we will explore the sensitivity of assuming an ongoing difference in readmission risk in a scenario analysis. We will also include the cost of other healthcare services, such as medicine use, and outpatient visits (both to the hospital and primary care) costed using standard Pharmaceutical Benefits Scheme/Medicare Benefits Scheme/hospital rates. Finally, we will include costs to patients and carers of receiving care, with a tailored questionnaire designed to capture costs associated with travel, parking, and employment losses. This can be used to estimate a societal economic evaluation

Regarding outcomes, we will first present cost per readmission avoided, reflecting our proposed primary outcome. We will then generate a cost-utility analysis through combination of the cost data described above with information around mortality and quality of life (captured using the EQ-5D-5L [53]) to generate a cost per quality-adjusted life year (QALY) of the package relative to usual care. Standard univariate and probabilistic sensitivity analysis will be conducted to identify key drivers of the results of the economic evaluation, and to suggest factors to which the result is most sensitive and hence appropriate for future research.

7. Acceptability and fidelity of the multifaceted intervention [54] gathered through semi-structured interviews with participating clinicians up to three months post completion of intervention. We will incorporate a thorough process evaluation to assess how the intervention was delivered to provide policymakers and practitioners with vital information about how the intervention could be replicated. A process evaluation also provides generalisable knowledge on how to implement complex interventions [54]. An acceptability framework survey will be used to obtain feedback from clinicians on the intervention [55, 56]. We will also purposively select clinicians for semi-structured interviews for qualitative feedback, with participants selected to achieve maximum variation (profession, role, gender, hospital or clinic). Issues explored through the survey and interviews will include:
  ◦ Overall acceptability for clinicians
  ◦ Burden (i.e. reasons given for discontinuation and/or dropout)
  ◦ Ethical consequences (i.e. associated side effects with intervention)
  ◦ Experience (i.e. user experience, user perceptions, satisfaction)
  ◦ Affective attitude (i.e. attitude towards intervention, attitude measures)
  ◦ Opportunity costs (i.e. influence on adherence, and participation)
  ◦ Intention (i.e. willingness to participate in the intervention)
  ◦ Perceived behavioural control (i.e. the extent to which the individual believes they have autonomy/control over the situation wherein the intervention takes place)
  ◦ Perceived treatment control (i.e. the extent to which the individual believes the treatment will be effective in curing the illness/helping the patient)

### Data analysis

Phase 1 and 2 qualitative data will be thematically analysed [57, 58]. Audio recordings will be professionally transcribed verbatim by an independent party and the transcripts quality screened and cross-referenced. The NVivo© software (QSR International Pty Ltd, Chadstone, Australia) will be used for coding by two coders. Where there are differing interpretations, the differences will be discussed between the members of the research team until consensus is reached. Data will be thematically analysed to identify underlying themes.

All phase 3 participants (excluding those recruited in the lead-in phase) will be included in the analysis. Each cluster will be classified as being in the intervention or the control phase based on their pre-specified randomised crossover time, regardless of whether crossover is achieved at that time. In the primary analysis, overall differences in readmissions will be modelled using a mixed effects Poisson regression model with a random effect for cluster (pharmacy) and a fixed effect for each step to account for any secular trend. Secular trends may include seasonal variation in readmissions or changes in practice (outside of the project’s control). We also intend to allow for both levels of clustering at the analysis stage, i.e. we will allow for both clustering by hospital and clustering by pharmacy (the unit of randomisation). This will be achieved by including both a random effect for community pharmacy hub and a random effect for hospital. Robust standard errors will be used to allow for the misspecification of the error structure when using the Poisson model to model binary events. We will report treatment effects both on the relative and absolute scale. We will also report estimates of intra cluster correlations. Secondary analysis will be conducted using similar techniques. The primary and secondary outcomes will be considered significant at the 5% level, and 95% confidence intervals will be reported. Missing outcome data is likely to be minimal for this study as it is routinely collected through hospital records. If the level of missing patient characteristic data is above 5%, we will use multiple imputation methods which allow for clustering and time effects. We will not conduct an interim analysis.

Development of phase 3 patient telephone surveys, clinician interview guides, and project data collection forms at hospital and community pharmacy levels will be guided by the Consolidated Framework for Implementation Research [59, 60]. The framework will be used to guide data collection, coding, analysis, and reporting of findings to obtain insights into the fidelity of implementation of each component of the intervention, including interprofessional communication and patient and health professional satisfaction with service(s) provided. The COREQ checklist and qualitative research criteria will be used in the development, analysis, and reporting of the phase 1 qualitative workshop data and phase 3 interviews with clinicians and patients [61, 62].

## Discussion

Stakeholders and end-users will be engaged to the co-design of a multifaceted intervention that will follow the patient’s transition of care, thus placing the patient at the centre of care. The proposed intervention will utilise transition of care strategies already in place to send discharge medicine handover information to GPs whilst introducing a risk stratification process to determine the medicine information handover to community pharmacies. There will be a pharmacist navigator at each of the trial hospitals who will be an experienced clinical pharmacist, embedded at hospital sites and with access to patients’ medical records, to facilitate handover to primary care clinicians.

The evaluation of the post-discharge medication review service in community pharmacies will provide insights into this model of care for national roll-out, as was the case in the UK [63, 64]. The digital solution and information technology capability will be translatable to other service areas, including the primary care management of patients with chronic conditions and other professional pharmacy services to enable seamless interdisciplinary team-based care.

## Trial status

This trial is registered with the Australian New Zealand Clinical Trials Registry: ACTRN12624000480583p, registered 19 April 2024, https://www.anzctr.org.au/ACTRN12624000480583p.aspx. Phase 1 recruitment commenced June 2024; phase 3 recruitment will be completed by 30 September 2026.

## Trial governance

The University of Queensland is the Primary Sponsor of the trial. Secondary Sponsors are partner organisations GCHHS, MSHHS, Monash University, Sydney University, Curtin University, Bond University, and the Pharmaceutical Society of Australia. Our steering committee consists of four CIs (CI Hattingh, CI Baysari, CI Foot, CI Morgan), one representative from each partner organisation, and includes key stakeholders such as hospital site representatives and a nominee from the consumer reference group. The steering committee provides technical advice on the project design, implementation, and analysis into the operational unit and project leadership group. Meetings are every 4 months. Any proposed changes to the project protocol will be discussed with the steering committee and if supported will be communicated to the Medical Research Future Fund as the funding agency and the primary and secondary sponsors whilst going through the ethics amendment process.

The safety committee will be six-member a group independent from the research team to identify and make recommendations on project safety issues throughout the project. Members will be recruited from the GCHHS and MSHHS Human Research Ethics Committees. The safety committee will conduct monthly trial audits and report on harms potentially associated with the intervention, e.g. if a patient’s nominated community pharmacy does not provide the intended MMR service and the patient suffers medication-related harm. The safety committee will provide advice to the project leadership group. This committee will meet every 4 months or more regularly as needed throughout phase 3.

The primary author of this manuscript is the contact for public and scientific queries. Deidentified trial participant-level data will not be shared.

## Dissemination

The results of this study will be published in peer-reviewed journals, locally through university and hospitals’ publicity channels, and be presented at academic conferences. We will follow the International Committee of Medical Journal Editors rules for authorship.

## Supplementary Information

Supplementary Material 1.

## Data Availability

All trial data will be deidentified and stored securely at GCHHS for the required period of 15 years from the end of the trial. Any data required to support the protocol can be supplied on request to the corresponding author.

## Abbreviations

GCHHS: Gold Coast Hospital and Health Service
GP: General practitioner
ieMR: Integrated electronic Medical Record
MMR: Medication management review
MRH: Medication-related harm
MSHHS: Metro South Hospital and Health Service
PSA: Pharmaceutical Society of Australia
SW-CRT: Stepped wedge cluster randomised controlled trial
WHO: World Health Organisation
