# Correction: OPTimising MEDicine information handover after Discharge (OPTMED-D): protocol for development of a multifaceted intervention and stepped wedge cluster randomised controlled trial

**DOI:** 10.1186/s13063-024-08528-5

**Published:** 2024-11-08

**Authors:** Laetitia Hattingh, Melissa T. Baysari, Holly Foot, Tin Fei Sim, Gerben Keijzers, Mark Morgan, Ian Scott, Richard Norman, Faith Yong, Barbara Mullan, Claire Jackson, Leslie E. Oldfeld, Elizabeth Manias

**Affiliations:** 1 Allied Health Research, Gold Coast Health, Southport, QLD 4215 Australia; 2 School of Pharmacy, The University of Queensland, Brisbane, QLD 4102 Australia; 3 School of Pharmacy and Medical Sciences, Grifth University, Gold Coast, QLD 4222 Australia; 4 Biomedical Informatics and Digital Health, School of Medical Sciences, Faculty of Medicine and Health, The University of Sydney, Sydney, NSW 2050 Australia; 5 Curtin Medical School, Curtin University, Bentley, WA 6102 Australia; 6 Emergency Department, Gold Coast Health, Southport, QLD 4215 Australia; 7 Faculty of Health Sciences and Medicine, Bond University, Gold Coast, QLD 4229 Australia; 8 Metro South Digital Health and Informatics, Princess Alexandra Hospital, Woolloongabba, QLD 4102 Australia; 9 School of Population Health, Curtin University, Bentley, WA 6102 Australia; 10 Rural Clinical School, Faculty of Medicine, The University of Queensland, Brisbane, QLD 4350 Australia; 11 Academy of Medical Education, Faculty of Medicine, The University of Queensland, Brisbane, QLD 4006 Australia; 12 The Westmead Institute for Medical Research, The University of Sydney, Sydney, NSW 2145 Australia; 13 General Practice and Primary Care Reform, The University of Queensland, Brisbane, QLD 4072 Australia; 14 School of Nursing and Midwifery, Monash University, Melbourne, VIC 3800 Australia

**Correction** **:** **Trials 25, 601 (2024)**

**https://doi.org/10.1186/s13063-024-08496-w**

Following the publication of the original article [1], we were notified that Figure 5 still had track changes. These have now been removed.

The original article has been corrected.
